# Supplementary material for: Magnetic properties of optimized cobalt nanospheres grown by focused electron beam induced deposition (FEBID) on cantilever tips
Source: Beilstein J Nanotechnol. 2017 Oct 9;8:2106–15. doi: 10.3762/bjnano.8.210 (PMC5647723; doi:10.3762/bjnano.8.210)
Supplement: File 1 — Additional experimental information. Chemical compositional maps from STEM-EELS and electron holography experiments have been performed on a 90 nm diameter sphere following the procedure described in the main text. These results are described and illustrated in this file. [file Beilstein_J_Nanotechnol-08-2106-s001.pdf]

Supporting Information

for

Magnetic properties of optimized cobalt nanospheres grown by focused electron beam induced deposition (FEBID) on cantilever tips

Soraya Sangiao[1,2,*], César Magén[1,2,3,4], Darius Mofakhami[5], Grégoire de Loubens[5] and José María De Teresa[1,2,4]

Address: [1]Laboratorio de Microscopías Avanzadas (LMA), Instituto de Nanociencia de Aragón (INA), Universidad de Zaragoza, 50018 Zaragoza, Spain, [2]Departamento de Física de la Materia Condensada, Universidad de Zaragoza, 50009 Zaragoza, Spain, [3]Fundación ARAID, 50018 Zaragoza, Spain, [4]Instituto de Ciencia de Materiales de Aragón (ICMA), CSIC, Universidad de Zaragoza, 50009 Zaragoza, Spain and [5]Service de Physique de l'Etat Condensé, CEA, CNRS, Université Paris-Saclay, 91191 Gif-sur-Yvette, France

* Corresponding author

Email: Soraya Sangiao* - sangiao@unizar.es

Additional Experimental Information

Chemical composition maps by STEM-EELS and electron holography experiments have been performed in the sphere with a diameter of 90 nm following the exact same procedure described in the main text, and are illustrated in Fig. S1 and Fig. S2. In the case of the holography results, a vortex state is also observed in the smallest sphere. The quality of the reconstruction of the magnetic induction fields is negatively affected by the presence of the tip inside the nanosphere and surface contamination. For instance, the uncertainty in the determination of the absolute magnetic phase shift image due to the rising contamination during the experiment increases as the size of the magnetic object decreases. Thus, the absolute values of the magnetization are less reliable. Indeed, the magnetic induction values obtained for the 90-nm-diameter sphere is higher than in the 110-nm one, which is not plausible and should be attributed to artifacts.

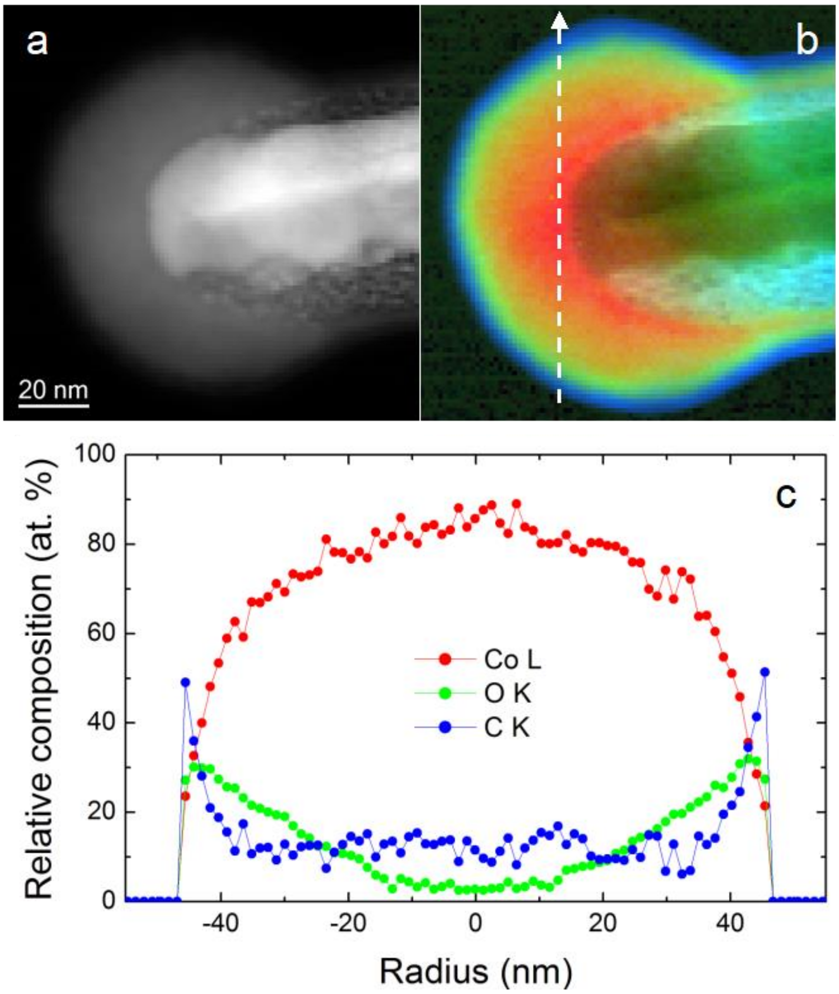

Figure S1: STEM-EELS compositional analysis of the cobalt nanosphere with 90 nm of diameter. (a) Reference image in Z contrast. (b) Colored chemical map, including the relative compositions of Co (red), O (green) and C (blue). (c) Compositional line profile extracted along the white arrow in (b).

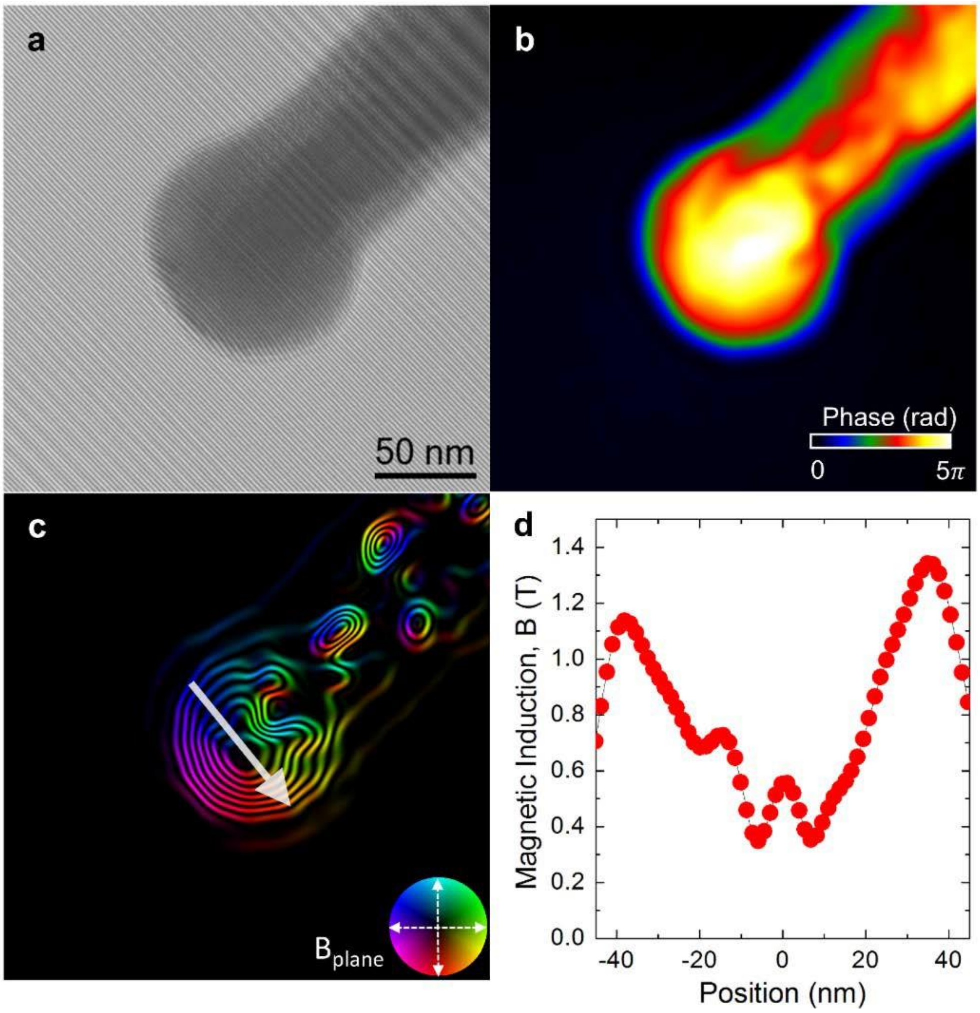

Figure S2: Electron holography of one cobalt nanosphere with a diameter of 90 nm. a) Electron hologram of the object. b) Electrostatic phase image. c) False colored representation of the magnetic induction flux lines. The inset represents the color scale of the magnetic induction orientation in arbitrary units, where the position of a color relative to the center of the circle corresponds to the orientation of the magnetic induction. d) Profile of the modulus of the magnetic induction vector as measured along the white arrow in c), where the position reference is taken at the minimum of the magnetic induction.
